# SC2disease: a manually curated database of single-cell transcriptome for human diseases

**DOI:** 10.1093/nar/gkaa838

**Published:** 2020-10-03

**Authors:** Tianyi Zhao, Shuxuan Lyu, Guilin Lu, Liran Juan, Xi Zeng, Zhongyu Wei, Jianye Hao, Jiajie Peng

**Affiliations:** School of Computer Science, Northwestern Polytechnical University, Xi’an 710072, China; Department of Physiology and Pathophysiology, School of Basic Medical Sciences, Xi’an Jiaotong University Health Science Center, Xi’an 710061, China; School of Computer Science, Northwestern Polytechnical University, Xi’an 710072, China; Department of Computer Science, Harbin Institute of Technology, Harbin, Heilongjiang 150001, China; School of Computer Science, Northwestern Polytechnical University, Xi’an 710072, China; School of Data Science, Fudan University, Shanghai 200433, China; School of Software, Tianjin University, Tianjin 300072, China; School of Computer Science, Northwestern Polytechnical University, Xi’an 710072, China

## Abstract

SC2disease (http://easybioai.com/sc2disease/) is a manually curated database that aims to provide a comprehensive and accurate resource of gene expression profiles in various cell types for different diseases. With the development of single-cell RNA sequencing (scRNA-seq) technologies, uncovering cellular heterogeneity of different tissues for different diseases has become feasible by profiling transcriptomes across cell types at the cellular level. In particular, comparing gene expression profiles between different cell types and identifying cell-type-specific genes in various diseases offers new possibilities to address biological and medical questions. However, systematic, hierarchical and vast databases of gene expression profiles in human diseases at the cellular level are lacking. Thus, we reviewed the literature prior to March 2020 for studies which used scRNA-seq to study diseases with human samples, and developed the SC2disease database to summarize all the data by different diseases, tissues and cell types. SC2disease documents 946 481 entries, corresponding to 341 cell types, 29 tissues and 25 diseases. Each entry in the SC2disease database contains comparisons of differentially expressed genes between different cell types, tissues and disease-related health status. Furthermore, we reanalyzed gene expression matrix by unified pipeline to improve the comparability between different studies. For each disease, we also compare cell-type-specific genes with the corresponding genes of lead single nucleotide polymorphisms (SNPs) identified in genome-wide association studies (GWAS) to implicate cell type specificity of the traits.

## INTRODUCTION

Single-cell RNA sequencing (scRNA-seq) technologies enable the study of the transcriptomic profile of complex multicellular organisms at single-cell resolution. This provides scientists with a new tool to investigate cellular heterogeneity in expression patterns (1), and in particular, the heterogeneity of disease cells. More importantly, the rapid development of scRNA-seq brings insight on exploring cellular subpopulations in the disease microenvironment, which is conducive to the study of disease occurrence, development, drug resistance (2) and immune escape (3).

The scRNA-seq technology has been applied in identifying differentially expressed genes in case control studies, and identifying differences between cell subpopulations (4,5). Many researchers have identified specificity of gene expression in diseases using scRNA-seq, such as identifying differentially expressed genes of multiple neuronal cell subgroups in Alzheimer ’s disease (6), characterizing molecular signatures of cancer stem cell subpopulations in different stages of chronic myeloid leukemia (7), and revealing cell type specific expression changes in type 2 diabetes (8), among other examples.

With the wide application of scRNA-seq in transcriptome profiling, several scRNA-seq-related databases have been developed. CellMarker (9) records 13,605 cell markers in hundreds of cell types in both humans and mice. PanglaoDB (10) provides a web server to visually display the clustering results and gene expression ranks of scRNA-seq experiments in mice and humans. scRNASeqDB (11) collects the ranks of gene expression in different cell types from 36 datasets in the Gene Expression Omnibus (GEO). SCPortalen (12) integrates single-cell metadata, cell images and sequence information, but is more focused on the technical properties of scRNA-seq data. SCDevDB (13) focuses on single-cell gene expression profiling in different developmental pathways, including 10 human scRNA-seq datasets. JingleBells (14) offers scRNA-seq binary sequence alignment/map (BAM) files about immune-related datasets for visualization of reads. Although these databases all provide researchers with resources for studying gene expression in different cell types and tissues at the cellular level, none of them have collected data about gene expression specificity in different disorders. As researchers have increasingly explored the functional heterogeneity of cancer cells through scRNA-seq technology, Yuan *et al.* (15) developed CancerSEA to provide information on differentially expressed genes in various cancers with multiple functional states. However, CancerSEA only focuses on cancers and genes’ correlation with these cancers, but does not provide expression information for each gene in specific cell types and tissues. Important information not contained in CancerSEA, such as average expression and fold change of expression in different pathologies, could help researchers find cell markers, differences in gene expression in different cell types and causal genes of diseases. Therefore, a database which collects expression of cell-type-specific genes in various human diseases is needed for researchers to further explore pathogenesis.

We have developed the SC2disease database, which focuses on providing differences in gene expression between pathological cases and healthy controls, between different cell types in pathological cases, and between cases with differing degrees of pathology. The SC2disease database provides a user-friendly interface for browsing the expression of various genes of interest, searching cell-type markers, exploring biomarkers of multiple diseases, comparing the expression profiles of various cell types in disease and non-disease states, and comparing scRNA-seq based results with GWAS studies. Overall, SC2disease, which is freely available (http://easybioai.com/sc2disease/), can serve as a comprehensive resource for users to explore gene expression specificity in different cell types, tissues, and diseases.

## DATA COLLECTION AND DATABASE CONTENT

**Figure 1.**
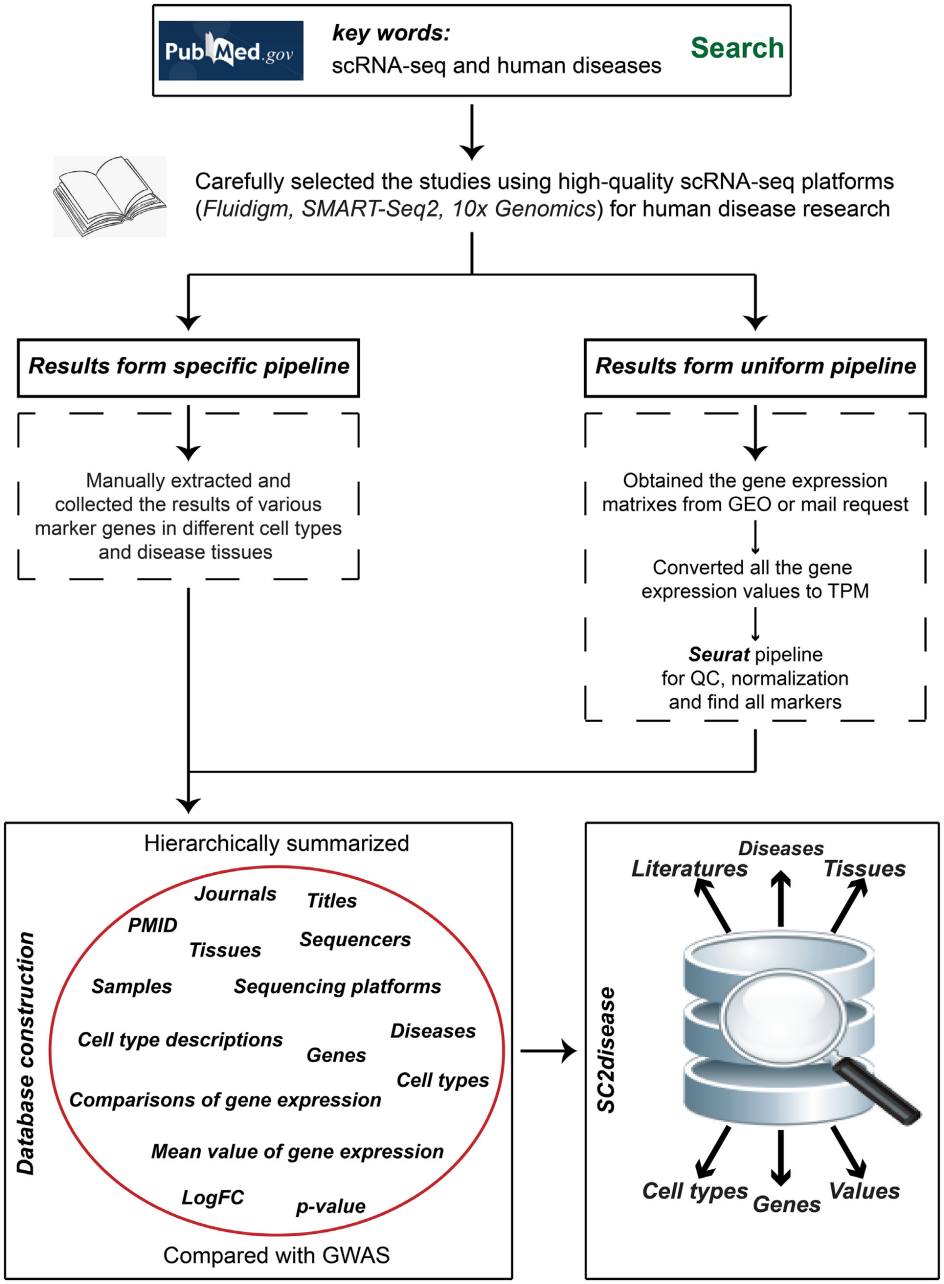
The process of data collection. We selected the studies using high-quality scRNA-seq platforms for human disease. The original authors of these literatures have developed their specific pipeline for analysing their raw data so we manually extracted the results of their cell-type-specific genes into SC2disease. In addition, to to improve the comparability between different studies, we designed a unified pipeline to reanalyze the gene expression matrix of each study. We also put these reanalyzed results into SC2disease. Finally, these cell-type-specific genes and their related information constructed SC2disease.

Cell-type-specific genes and their expression in human diseases were manually extracted from publications. These publications were obtained from the PubMed database by searching for key words such as ‘single cell sequencing’, ‘single cell sequencing disease’, and ‘10× genomics’. Subsequently, their corresponding human diseases, experimental tissues, cell types, significant genes and expression were extracted and double checked. The data collection process is shown in Figure 1.

**Figure 2.**
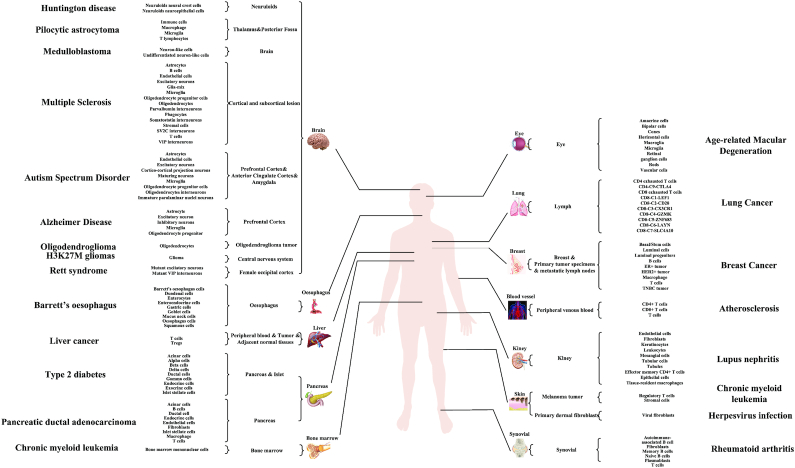
SC2diseases collects 25 human diseases-related cell-type-specific genes obtained by scRNA-seq. All the cells were extracted from 29 tissues and classified into 341 cell types.

Finally, the expression of genes in 341 cell types and 29 tissues which are related to 25 diseases were collected in the current version of SC2disease. These diseases and their experimental tissues and cell types are shown in Figure 2.

A total of 946 481 entries were recorded in SC2disease. Each entry contains 10 sections for describing the relationship between a gene and the relevant disease. The 10 sections include name of disease, experimental tissue, cell type, name of gene, variable names used to describe gene expression (log_2_FC or mean), the value of the variable, differentially expressed gene (DEG) comparison, an identifier for the source publication, sequencing platform and details. The ‘details’ section consists of detailed information on cell type, disease and gene. For ‘cell type,’ the cell's function is described. For ‘disease,’ its disease ontology (DO) ID (16), Medical Subject Headings (MeSH) ID (17) and description are given. For ‘gene,’ the detailed information includes gene symbol, EntrezID (18), involved pathway in KEGG (19), ID of the corresponding encoding protein in UniProt (20), its location in the genome, the the full name of the gene.

**Figure 3.**
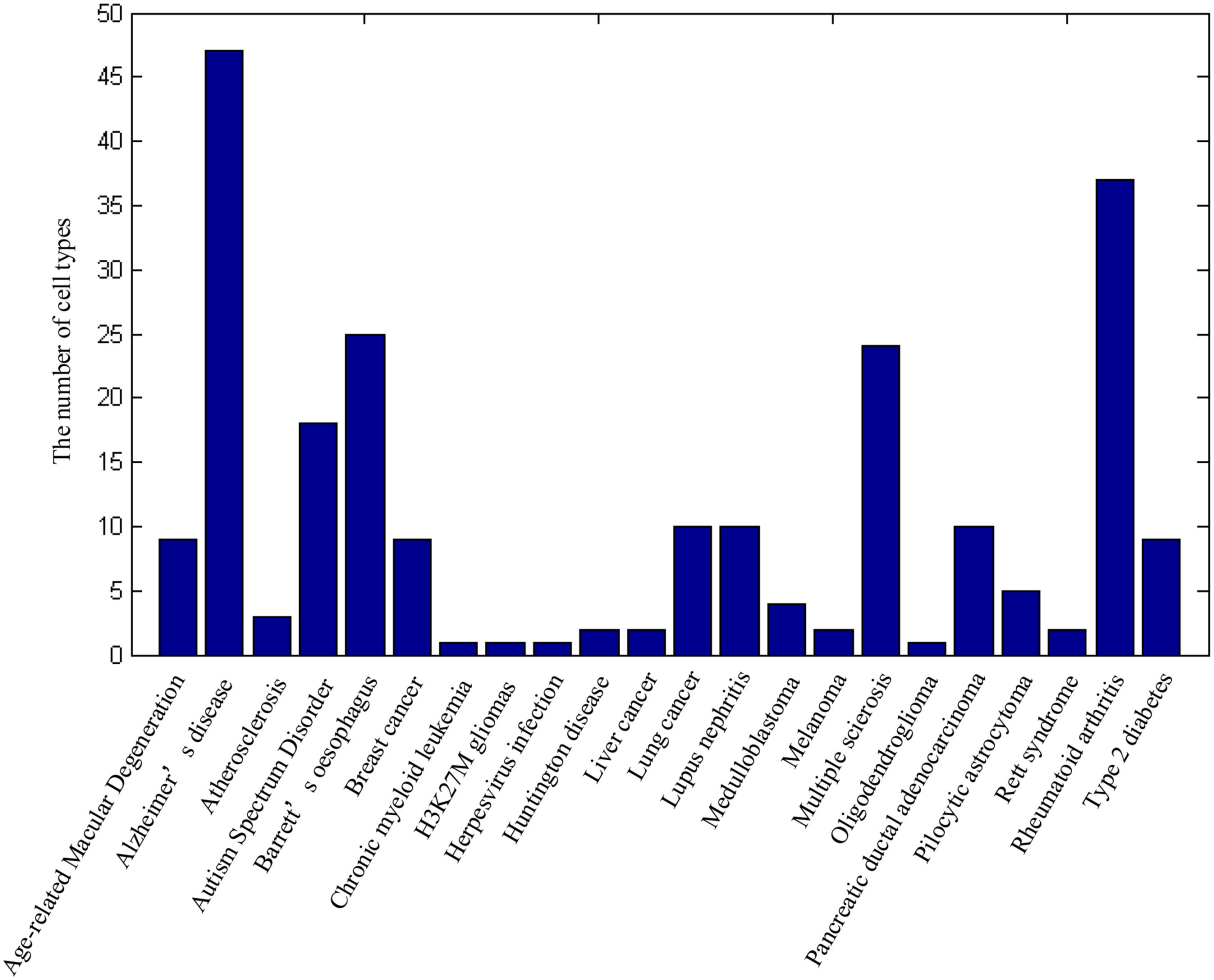
The number of cell types for each disease.

Figure 3 shows a histogram of the number of cell types for each disease. Due to the different experimental tissues used in each study, the number of cell types in different diseases vary. However, some studies did not cluster cells, so some diseases only have one cell type.

## USER INTERFACE

**Figure 4.**
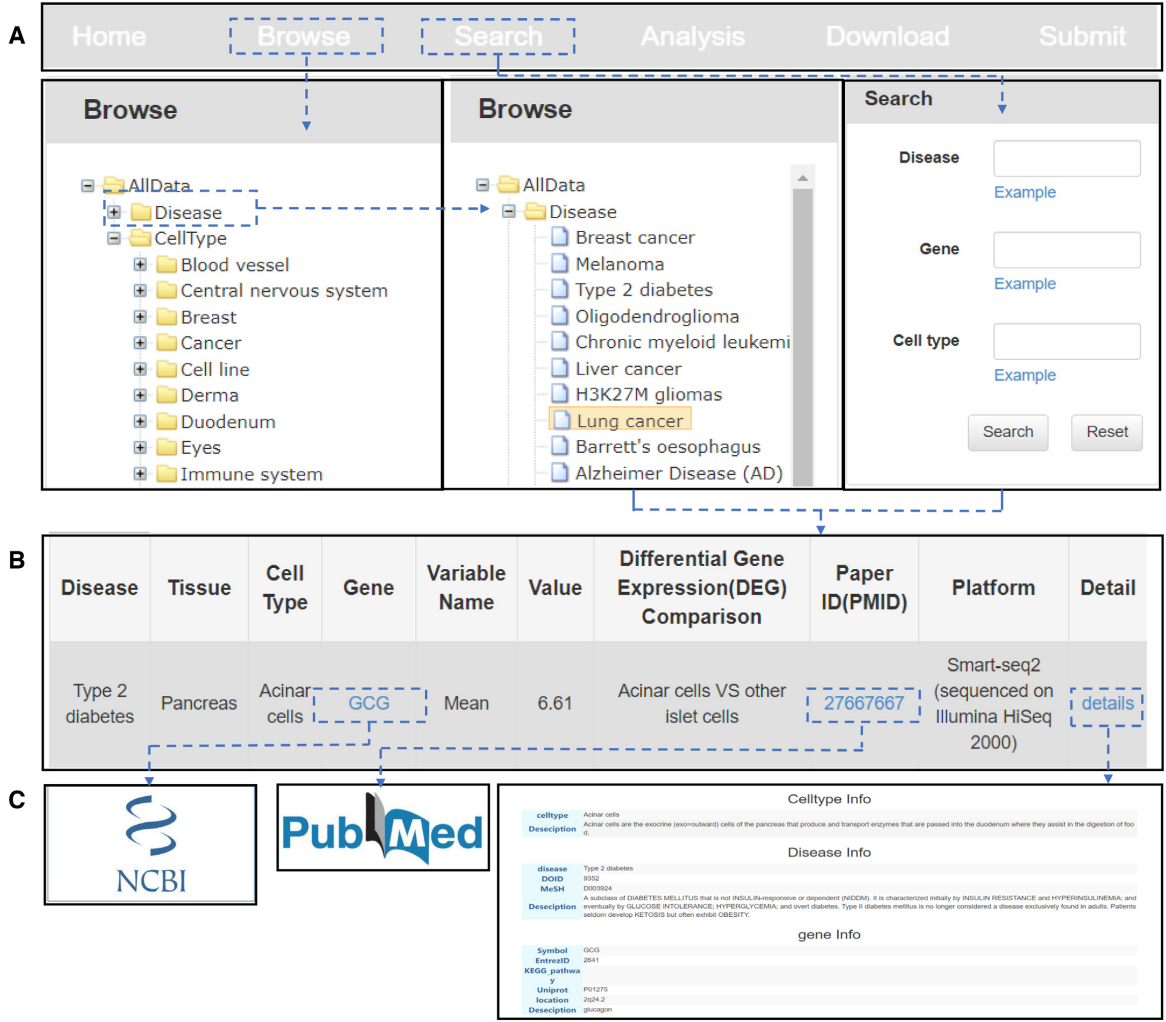
Schematic workflow of SC2diseases.

SC2disease provides a tree browser and a search engine to query detailed information about cell-type-specific genes in different diseases. Figure 4 shows the schematic workflow.

‘Disease’ and ‘Cell Type’ are the root categories of the SC2disease tree browser. A total of 25 types of diseases are included in the ‘Disease’ root category and detailed information of cell-type-specific genes which are related to the disease of interest are shown by clicking the name of the disease. We initially classified all cell types into 16 groups to help researchers who want to browse for marker genes in specific cell types. Researchers can also search their diseases, genes or cell types of interest by using the ‘Search’ function. For example, if we click on our disease of interest, ‘type 2 diabetes’, a list of cell-type-specific genes would be retrieved and shown as Figure 4B. The information on each gene will be shown as a line in the table, which includes the name of the disease, the experimental tissue, the cell type, the name of the gene, variable names used to describe gene expression (log_2_FC or mean), the value of the variable, the DEG comparison, an identifier for the source publication, the sequencing platform and details. As shown in Figure 4C, by clicking the name of the gene, the link of this gene in NCBI will pop up. We can also click the ‘paper ID’ to explore more detailed information in the original literature. Finally, we also summarize detailed information on the disease, cell type and gene in the ‘details’ section.

SC2diseases also provides a ‘Download’ function for researchers accessing the whole dataset. In addition, the ‘Submit’ page was developed to offer other researchers a convenient way to upload new data that are not recorded in SC2disease.

**Figure 5.**
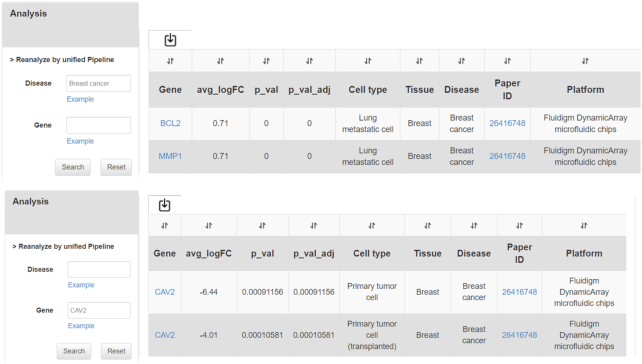
The results obtained by reanalyzing the gene expression matrix of the literature through the unified pipeline.

In addition to the above functions, to improve the comparability between different studies, we designed a unified pipeline to reanalyze the gene expression matrix of each study. Our pipeline includes two parts: first, we converted the value of gene expression (read counts, RPKM, etc.) into TPM (Transcripts Per Million); second, we used the R package Seurat (21) to do the downstream analysis, including quality control, normalization, gene expression comparison. It is noted that we excluded the genes which expressed in less than three cells. The cells with <200 genes expressed and >5% mitochondrial RNA. Then, we normalized and scaled the data by Seurat functions NormalizeData and ScaleData, respectively. Finally, we generated the results of marker genes of all cell types by Seurat function FindAllMarkers. Users could access reanalyzed data in the ‘analysis’ interface. Figure 5 shows an example to use this function.

As shown in Figure 5, users can search their interested diseases or genes by disease names or gene symbols in the left dialog boxes. The reanalyzed data would be shown in the right side as a table.

**Figure 6.**
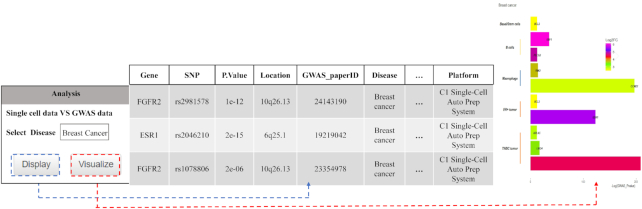
Susceptibility genes of diseases shared by single-cell-based result and GWAS-based result.

SC2disease also provides the susceptibility genes of diseases derived from both single-cell-based results and GWAS-based results. All the GWAS data were obtained from the GWAS catalog (22). Figure 6 shows the way to achieve this function. Taking ‘type 2 diabetes’ as an example, the list of susceptibility genes which are detected by both scRNA-seq and GWAS would be shown by clicking ‘Visualize’. In addition, the results could be displayed visually by clicking ‘Visualize’. In the resulting figure, the x-axis is the minimum *P* value of SNPs in the gene which were obtained from the GWAS results. The y-axis is the cell types of the overlapping susceptibility genes for a disease which were obtained from scRNA-seq results. The colors of the bars show the log_2_-fold change in gene expression.

## DISCUSSION

SC2disease is a comprehensive resource for documenting cell-type-specific genes of human diseases, which provides an easy way to search, browse, and download all the summarized results of scRNA-seq. SC2disease mainly has three advantages. First, SC2disease is the first resource of cell type-specific genes related to human diseases based on scRNA-seq. Second, we re-analyzed the gene expression matrix to make cell type-specific genes comparable between different diseases. Third, we also provide the results of both GWAS and scRNA-seq, which is convenient for researchers to explore the mechanism of gene pathogenesis. Since SC2disease is the first manually curated resource for collecting cell-type-specific genes of human diseases based on scRNA-seq, with the development and application of scRNA-seq technology, SC2disease will continue to be enriched and expand, which will help researchers understand the pathogenesis of human diseases.
